# *Psechrus kunmingensis*: description of male and supplementary description of female, with discussion on intraspecific variation (Araneae, Psechridae, *Psechrus*)

**DOI:** 10.3897/zookeys.238.3388

**Published:** 2012-11-07

**Authors:** Ping Feng, Yan Yan Ma, Zi Zhong Yang

**Affiliations:** 1 Key Laboratory of Forest Disaster Warning and Control in Yunnan Province, Southwest Forestry University, Kunming, 650224, China; 2 College of Agronomy and Bioscience, Dali University, Dali, Yunnan Province, 671003, China

**Keywords:** Araneae, taxonomy, intraspecific variation, morphology

## Abstract

*Psechrus kunmingensis* Yin, Wang & Zhang, 1985 was first described from the female only. The first illustration of the male appeared without any text description and lacked other critical information. For this study, we collected fresh specimens of this species from diverse localities around Yunnan Province, China. Here, the male is described in detail for the first time and a supplementary description of the female is given. Based on the largest collection of *Psechrus kunmingensis* specimens ever assembled, we found a remarkably high level of morphological variation in this species.

## Introduction

Psechridae Simon, 1890 is a small spider family with 2 genera, 46 species (Bayer 2012). Among them, 14 species belonging to both genera are reported from China (Li and Wang 2012). The distribution of psechrids runs from southern China and South East Asia to Queensland, Australia (Platnick 2012). Up to now, there were three revisions on this family: Levi (1982) made a revision of all psechrids known at that time, Wang and Yin (2001) focused on psechrids in China, and recently Bayer (2011, 2012) respectively revised all species of *Fecenia* and *Psechrus*. In the genus *Psechrus*, several species are known from only one sex. *Psechrus borneo* Levi, 1982, *Psechrus kunmingensis* Yin, Wang & Zhang, 1985, *Psechrus jinggangensis* Wang & Yin, 2001, and *Psechrus kenting* Yoshida, 2009, for example, are known only from females. Many species of *Psechrus* vary a lot and it is very important to differentiate intraspecific variation from characters of different species. Levi (1982) illustrated variation of several species, but Bayer (2012) pointed out that Levi considered clearly differing structures of copulatory organs as intraspecific variation.

*Psechrus kunmingensis* was described and illustrated for the first time by Yin et al. (1985) based on 4 female specimens collected from Kunming, Yunnan Province, China. Later, Song et al. (1999) illustrated both the male and the female, but provided neither descriptions nor exact specimen collection locality or place of deposition. They gave no justification for their conclusion that the male they illustrated was conspecific with *Psechrus kunmingensis*. Wang and Yin (2001) redescribed and illustrated the female.

We examined specimens collected from the type locality of *Psechrus kunmingensis* (Kunming, Yunnan Province, China) at the same time of year (April to July) and many specimens from places around Kunming. Based on examination of these specimens, we concluded that the male illustrated by Song et al. (1999) is indeed conspecific with the *Psechrus kunmingensis* female. In the present paper, the male of *Psechrus kunmingensis* is described in detail. Additionally, a supplementary description of females is provided. We illustrate and describe a high level of morphological intraspecific variation for the first time, which will be important for further research and proper identification of this species. Some photographs generated in the course of this study were shared with Steffen Bayer and appeared with our permission in the recent taxonomic revision of *Psechrus* (Bayer 2012). Based on this, Bayer was able to include a brief description of the male palp of *Psechrus kunmingensis* in his monograph. In this paper, we are able to provide a more complete description of the male anatomy and coloration, measurement data, and a survey of intraspecific variation.

## Methods

Specimens were preserved in 75% ethanol. Female copulatory organs were dissected and cleared in 90% lactic acid for a few minutes. Photographs were taken with Nikon digital Sight DS-Fi1 mounted on Nikon SMZ1000 Stereoscopic Zoom Microscope. Copulatory organs were illustrated using Adobe Illustrator CS5, with a Wacom Bamboo CTL-660 pen and tablet device. Illustrations were rendered in Adobe Photoshop CS5 Extended.

All measurements are in millimeters (mm), and taken with Nikon NIS-Elements Imaging Software Br (version 2.34). All scale bars are 0.5mm length. We measured two male specimens and 5 female specimens to obtain size range data. Specimens were selected to cover the widest possible size range. The “prosoma length” or “opisthosoma length” respectively refers to length of the main part of prosoma or opisthosoma , without spinnerets and petiolus. The whole “body length” is regarded as length from clypeus to the posterior tip of opisthosoma. Every individual was given a code, consisting of abbreviation of the locality and a sequence number. For instance, “KM18” represents a specific specimen collected from Kunming. Non-quantitative descriptions of somatic morphology are based on KM37 (female) and KM38 (male). Palpal and leg spination pattern is given as: prolateral, dorsal, retrolateral, ventral (the latter digit may be omitted in the case of the absence of ventral spines) (Jäger and Kunz 2010). The term “subadult” female refers to specimens that possess only pre-epigynes (Bayer 2011). All material is deposited at College of Agronomy and Bioscience, Dali University, Yunnan, China, except the following specimens, which are deposited at Senckenberg Museum, Frankfurt am Main, Germany (SMF): 1 male (KM04), one female (KM07) and one subadult female (KM25).

### Abbreviations

ALE–anterior lateral eyes, AME–anterior median eyes, PLE–posterior lateral eyes, PME–posterior median eyes; C–conductor, CB–cymbium, E–embolus, EA–embolic apophysis; CD–copulatory duct, CO–copulatory opening, FD–fertilisation duct, LL–lateral lobe, MS–median septum, SB–spermathecal base, SG–muscle sigilla, SH–spermathecal head, SO–slit sense organ; I, II, III, IV–legs I to IV.

## Taxonomy

### Psechrus kunmingensis

Yin, Wang & Zhang, 1985

http://species-id.net/wiki/Psechrus_kunmingensis

**Figures 1–9.**
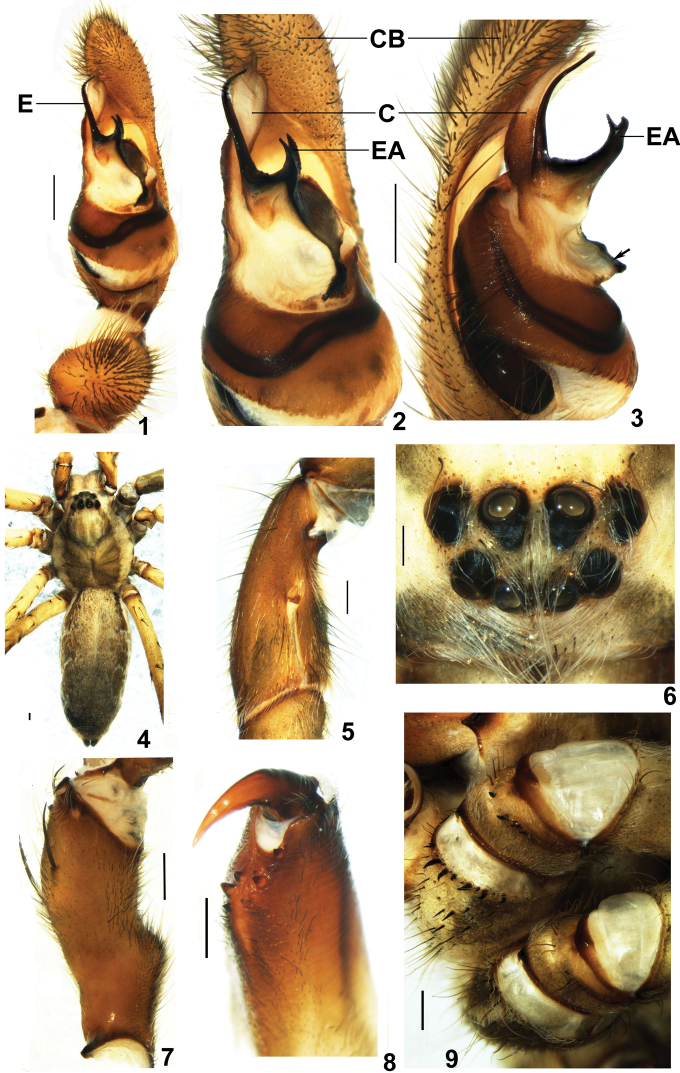
*Psechrus kunmingensis* Yin, Wang & Zhang, 1985. Male (KM18). **1–3** left palp (**1, 2** ventral **3** prolateral) **4** habitus, dorsal **5** left patella, retrolateral **6** eyes arrangement, dorsal **7** left femur , prolateral **8** left chelicera, retrolateral **9** left coxa I ventral. Arrow indicates a broad apophysis at embolic base. **C** conductor **CB** cymbium **E** embolus **EA** embolic apophysis (Scale bar 0.5mm).

Figs 1 [Fig F2] [Fig F3] [Fig F4] [Fig F5] –24

Psechrus kunmingensis Yin, Wang & Zhang 1985: 25, Fig. 5A–D (Description and illustration of female).
Psechrus kunmingensis Song, Zhu & Chen 1999: 397, Fig. 232C–D, O–P (Illustration of male and female).
Psechrus kunmingensis Wang & Yin 2001: 334, Figs 9–10 (Description and illustration of female).

#### Material examined.

China, Yunnan Province:Xi Shan, Kunming, 2260m, 24°57'24.3"N, 102°37'43.7"E: 4 ♂ (KM01-04), 6 ♀ (KM05-10), 27-IV-2004, Zi Zhong Yang leg.; 5 ♀ (KM11-15), 27-IV-2004, Zhi Sheng Zhang leg.; 1 ♂ (KM18), 5 ♀ (KM16-17, KM19-21), 2 subadults (KM22, KM25), 12 juveniles (KM23-24, KM26-35), 20-IV-2011, Ping Feng and Yan Yan Ma leg.; 1 ♀ (KM36), 07-IX-2011, Zi Zhong Yang leg.; 1 ♂ (KM38), 1 ♀ (KM37), 04-V-2011, Zong Xu Li leg.. Maguohe township (by the 101 provincial road), Malong county, Qujing, 1880m, 25°27'50.7"N, 103°22'42.8"E, 3 ♀ (ML01-03), 18-VII-2012, Ping Feng and Ting Bang Yang leg.. Dong Shan, Xuanwei, Qujing, ca 2150m, 26°12'42.0"N, 104°08'15.6"E, 10 ♀ (XW01-10), 7 juveniles (XW11-17), 17-VII-2012, Ping Feng, Yan Yan Ma and Ting Bang Yang leg.. Yongfeng town, Zhaotong, ca 1930m, 27°14'55.6"N, 103°40'10.6"E, 1 ♀ (ZT01), 23-VII-2004, Zi Zhong Yang leg.. Fenghuang Shan, Zhaotong, 1970m, 27°18'30.0"N, 103°42'16.7"E, 4 ♀ (ZT02-05), 14-VII-2012, Ping Feng and Yan Yan Ma leg.. Huaning county, Yuxi, ca 1660m, 24°10'59.3"N, 102°56'45.2"E, 1 ♀ (HN01), 09-VIII-2002, Jin Yin Lu leg.. Mopan Shan, Xinping county, Yuxi, ca 1630m, 24°01'21.3"N, 101°58'15.7"E, 2 ♀ (XP01-02), 05-V-2012, Zi Zhong Yang leg.. Lukou village (by the 204 provincial road), Luxi county, Honghe Autonomous Prefecture, 1600m, 24°28'30.2"N, 103°32'35.7"E, 3 ♀ (LX01-03), 1 juvenile (LX04), 27-VII-2012, Ping Feng and Ting Bang Yang leg.. Kaiyuan (by the 323 national road), Honghe Autonomous Prefecture, 1980m, 23°43'27.7"N, 103°23'05.9"E, 5 ♀ (KY01-05), 1 juvenile (KY06), 26-VII-2012, Ping Feng and Yan Yan Ma leg.. Baila Shan, Luoping conty, Qujing, 1710m, 24°52'20.1"N, 104°15'37.2"E, 1 ♀ (LP01), 19-VII-2012, Ping Feng and Yan Yan Ma leg.. Guizhou Province: Weining county (by the 326 national road), Bijie, 2330m, 26°51'44.0"N, 104°18'15.2"E, 1 ♀ (WN01), 15-VII-2012, Ping Feng, Yan Yan Ma and Ting Bang Yang leg.

#### Type records.

Kunming, Yunnan Province, China, 1 ♀ holotype, 5-IV-1979, Jia Fu Wang leg.; 3 ♀ paratypes, VII-1983, Ming Yao Liu leg.; 4 ♀, 21-VII-1981, Jia Fu Wang leg.

#### Diagnosis.

Maleresembles *Psechrus sinensis* Berland & Berland, 1914 (Wang & Yin 2001: 338, Figs 25–26), *Psechrus tingpingensis* Yin, Wang & Zhang, 1985 (Wang & Yin 2001: 340, Figs 31–33) and *Psechrus triangulus* Yang et al., 2003 (Yang et al. 2003: 45, Figs D-F) by having an embolic basal apophysis (Figs 1–3) and a rounded bulge with dense patch of hairs on the ventral palpal femur (Fig. 7); distinguished from these species by: 1) embolic basal apophysis long and distally bifurcated (Figs 1–3, 17–19); 2) much longer embolus (almost as long as the width of the tegulum) (Figs 2, 17); 3) distal section of embolus directed retrolaterally. Female copulatory organs similar to *Psechrus sinensis* (Wang & Yin 2001: 338, Fig. 28) and *Psechrus jinggangensis* Wang & Yin, 2001 (Wang & Yin 2001: 335, Fig. 12) by having coiled copulatory ducts; distinguished from these species by the posteriorly and laterally lobed epigynal median septum, which interlocks with lateral lobe, and folds inward; the spermathecal heads arise anteriorly at spermathecae (Figs 11, 24a, 24b).

**Figures 10–16. F2:**
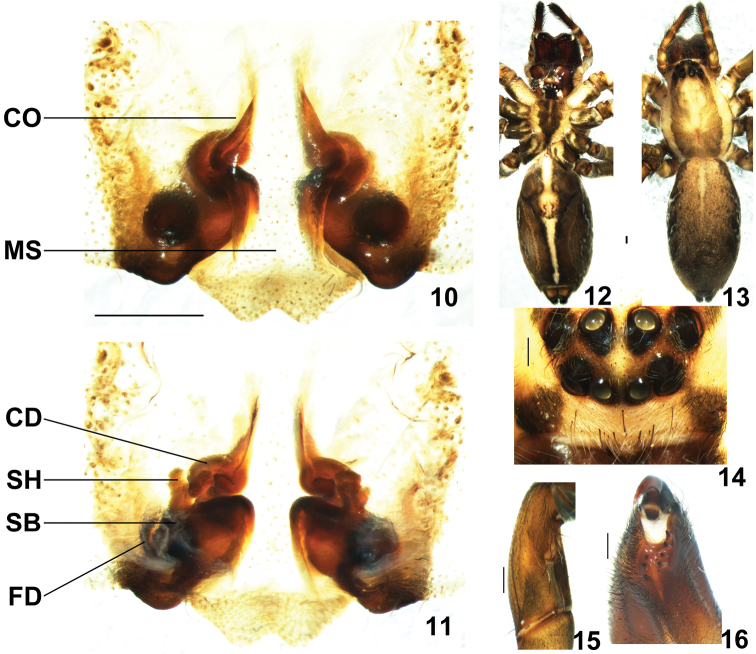
*Psechrus kunmingensis* Yin, Wang & Zhang, 1985. Female (KM37). **10** epigyne, ventral **11** vulva, dorsal **12–13** habitus (**12** ventral **13** dorsal,) **14** eyes arrangement, dorsal **15** left patella, retrolateral **16** left chelicera, ventral. **CD** copulatory duct **CO** copulatory opening **FD** fertilisation duct **MS** median septum **SB** spermathecal base **SH** spermathecal head (Scale bar 0.5mm).

**Figures 17–22. F3:**
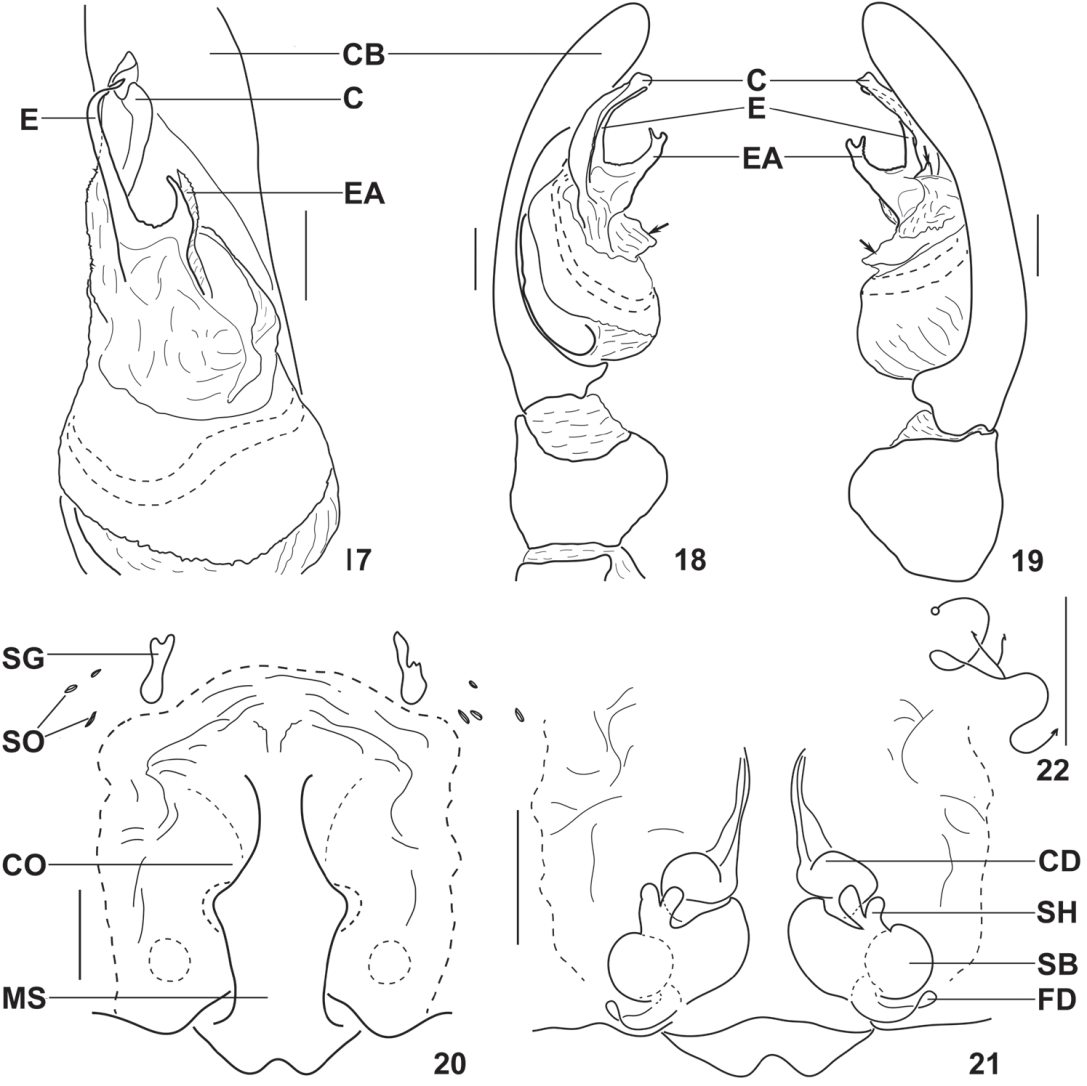
*Psechrus kunmingensis* Yin, Wang & Zhang, 1985. Copulatory organs. **17–19** male palp (**17** ventral **18** prolateral **19** retrolateral) **20–22** female copulatory organ (**20** epigyne, ventral **21** vulva, dorsal **22** Schematic course of internal duct system). Arrow in Fig. 18 and the lower one in Fig. 19 indicate the broad apophysis at embolic base; the upper arrow in Fig. 19 indicate the triangular apophysis besides embolus. **C** conductor **CB** cymbium **E** embolus **EA** embolic apophysis **CD** copulatory duct **CO** copulatory opening **FD** fertilisation duct **MS** median septum **SB** spermathecal base **SG** muscle sigilla **SH** spermathecal head **SO** slit sense organ (Scale bar 0.5mm).

#### Description.

Males (KM38 first, KM03 in parentheses): Body length 18.21 (15.05). Prosoma length 7.53 (6.46), prosoma width 5.89 (4.56), opisthosoma length 11.18 (7.37), opisthosoma width 5.08(2.93). Eyes diameter: AME 0.32 (0.34), ALE 0.39 (0.37), PME 0.35(0.37), PLE 0.43 (0.42). Distance between eyes: AME-AME 0.23 (0.18), AME-ALE 0.14 (0.08), ALE-PLE 0.53 (0.44), PME-PME 0.23 (0.25), PME-PLE 0.38(0.33), AME-PME 0.57 (0.56). Clypeus height at AME 0.85 (0.76), clypeus height at ALE 0.80 (0.69).

Carapace brown, with a gray band at central part and white hair along the margin (Fig. 4). Cervical groove and fovea with dark stripe (Fig. 4). Eight eyes arranged in two recurved rows, eye region with long white hairs (Fig. 6). Sternum light brown, with an inverted triangle dark mark and long hairs. Labium deep reddish brown; gnathocoxae brown. Chelicerae yellow at basal part, and reddish brown at terminal part, with 3 promarginal, 5 retromarginal teeth, and 3 denticles (Fig. 8). Legs yellow to reddish brown; Coxae and trochanteri of the first walking legs with short macrosetae in a distal row each (Fig. 9). Patellae of legs with a slit at retrolateral side (Fig. 5). Dorsal opisthosoma dark gray, with a pair of longitudinal black patches at lateral side, and pairs of white radiative patches. Ventral opisthosoma with a white band, from pedicel to cribellum.

**Figure 23. F4:**
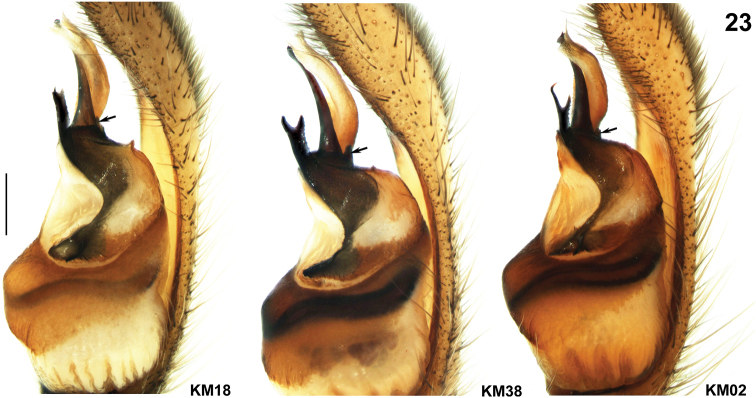
*Psechrus kunmingensis* Yin, Wang & Zhang, 1985. Intraspecific variation of male palp, retrolateral. Arrows indicate the triangular apophysis besides embolus (Scale bar 0.5mm).

Male palp: distal 1/3 part of dorsal palpal CB with dense scopula. Embolic base with a long and distally bifurcated apophysis (Figs 1–3, 17–19); conductor close to embolus, almost joined together along their entire lengths. In retrolateral view, there is a small triangular apophysis (as indicated by arrows) beside the embolus (Figs 19, 23); Tegulum with a broad apophysis (as indicated by arrows) under embolic base (Figs 3, 18–19). Sperm duct visible through tegulum, follows slightly meandering path ventrally (Figs 1, 2, 17). Palpal tibia very short, with dense patch of hairs at retrolateral side (Fig. 1); palpal femur with a rounded bulge and dense patch of hairs (Fig. 7).

Spination of palp and legs as shown in Table 1; Measurements of the palp and legs as shown in Table 2. KM38 first, KM03 in parentheses. Leg formula: 1423.

**Table 1.** Spination of palp and legs of *Psechrus kunmingensis* Yin, Wang & Zhang, 1985<br/>

**Male**	**Femur**	**Patella**	**Tibia**	**Metatarsus**
Palp	131	000	000	–
I	625	000	3038	4041
II	526	000	3036	3035
III	536	000	2033	3035
IV	435	000	3024	3035
**Female**	**Femur**	**Patella**	**Tibia**	**Metatarsus**
Palp	131	020(010 right)	012	2031
I	525	000	3037	2025
II	525	000	3036	2025
III	425	000	2034	3035
IV	543	000	2034	3045

**Table 2.** Measurements of palp and legs of *Psechrus kunmingensis* Yin, Wang & Zhang, 19850<br/>

**Male**	**Femur**	**Patella**	**Tibia**	**Metatarsus**	**Tarsus**	**Total**
Palp	2.94(2.81)	1.24(1.06)	1.08(0.96)	–	3.55(3.00)	8.81(7.83)
I	14.04(11.99)	3.70(3.00)	17.36(14.12)	16.18(13.07)	6.83(5.83)	58.11(48.01)
II	12.50(10.42)	3.21(2.76)	12.97(10.71)	12.58(10.33)	5.09(4.69)	46.35(38.91)
III	9.13(7.69)	2.72(2.25)	7.89(6.55)	8.55(7.13)	3.75(3.43)	32.04(27.05)
IV	12.80(10.27)	3.10(2.33)	12.98(10.30)	13.14(10.95)	5.60(5.30)	47.62(39.15)
**Female**	**Femur**	**Patella**	**Tibia**	**Metatarsus**	**Tarsus**	**Total**
Palp	2.74 (2.74–3.38)	1.18 (1.05–1.61)	1.32 (1.45–1.61)	–	2.29 (2.29–3.30)	7.53 (7.53–9.90)
I	10.11 (9.98–10.17)	3.27 (2.85–3.27)	11.52 (10.24–11.52)	9.41 (8.84–9.41)	5.11 (4.26–5.11)	39.42 (37.96–36.45)
II	8.31 (8.31–11.93)	2.78 (2.43–3.40)	9.06 (8.40–13.27)	7.61 (7.28–11.09)	4.23 (3.89–5.82)	31.99 (30.79–45.51)
III	6.94 (6.66–7.55)	2.28 (2.05–2.42)	5.93 (5.34–6.36)	5.33 (5.04–6.16)	3.33 (2.90–3.47)	23.81 (21.99–25.96)
IV	9.36 (8.91–10.57)	2.53 (2.08–2.90)	8.95 (8.28–9.50)	8.02 (7.84–8.81)	4.38 (4.15–4.38)	33.24 (31.26–36.07)

Females (KM37 first, together with those of others [KM05, KM06, KY02 and ZT04] given as ranges in parentheses): Body length 16.75 (14.17–20.30). Prosoma length 7.09 (5.15–7.50), prosoma width 5.07 (4.26–4.83), opisthosoma length 9.90 (8.52–13.46), opisthosoma width 5.08 (4.19–7.75). Eyes diameter: AME 0.33 (0.30–0.33), ALE 0.36 (0.44–0.47), PME 0.39(0.38-o.44), PLE 0.43 (0.41–0.47). Distance between eyes: AME-AME 0.20 (0.18–0.16), AME-ALE 0.11 (0.07–0.13), ALE-PLE 0.59 (0.42–0.55), PME-PME 0.29 (0.24–0.27), PME-PLE 0.34 (0.35–0.37), AME-PME 0.64 (0.55–0.53). Clypeus height at AME 0.75 (0.72–0.83), clypeus height at ALE 0.72 (0.71–0.81).

Coloration (Figs 12–13) as in male only generally darker; other characters as in male except as noted. Chelicerae with 3 promarginal, 5 retromarginal teeth, and 4 denticles (Fig. 16). Patellae of legs with a slit at retrolateral side (Fig. 15).

Female copulatory organ: the SO and SG are outside the epigynal field (the SO are anterior and lateral to the epigyne, while the SG are right anterior to the epigyne); the MS lobed at the posterior and lateral edges; there are many wrinkles at the epigyne, especially at the anterior part (Fig. 20); the shadow of the round spermathecae is evidently visible through the ventral view of epigyne (Fig. 10). Copulatory ducts coiled; the spermathecae is mostly covered by folds of LL and MS; the spermathecal heads arise anteriorly at spermathecae (Figs 11, 21). The incision at posterior margin of MS, shape of lobes of MS, the length of CD and shape of SH vary a lot (Fig. 24a, b); schematic course of internal duct system shown in Fig. 22.

**Figure 24a. F5:**
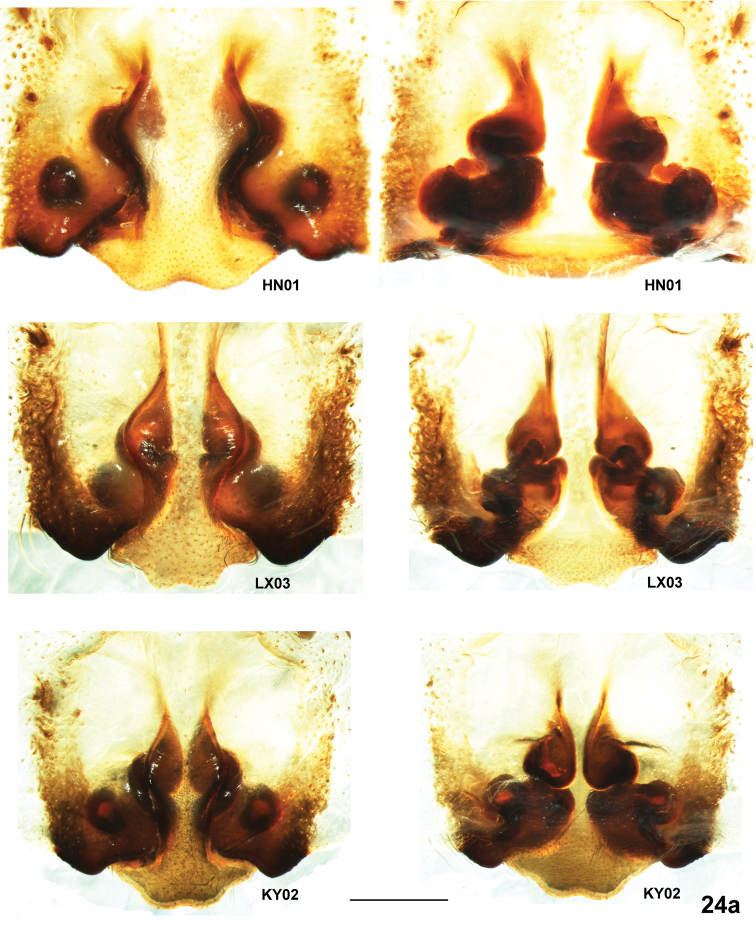
*Psechrus kunmingensis* Yin, Wang & Zhang, 1985. Intraspecific variation of female copulatory organ (left row, epigyne, ventral; right row, vulva, dorsal). (Scale bar, 0.5mm).

**Figure 24b.**
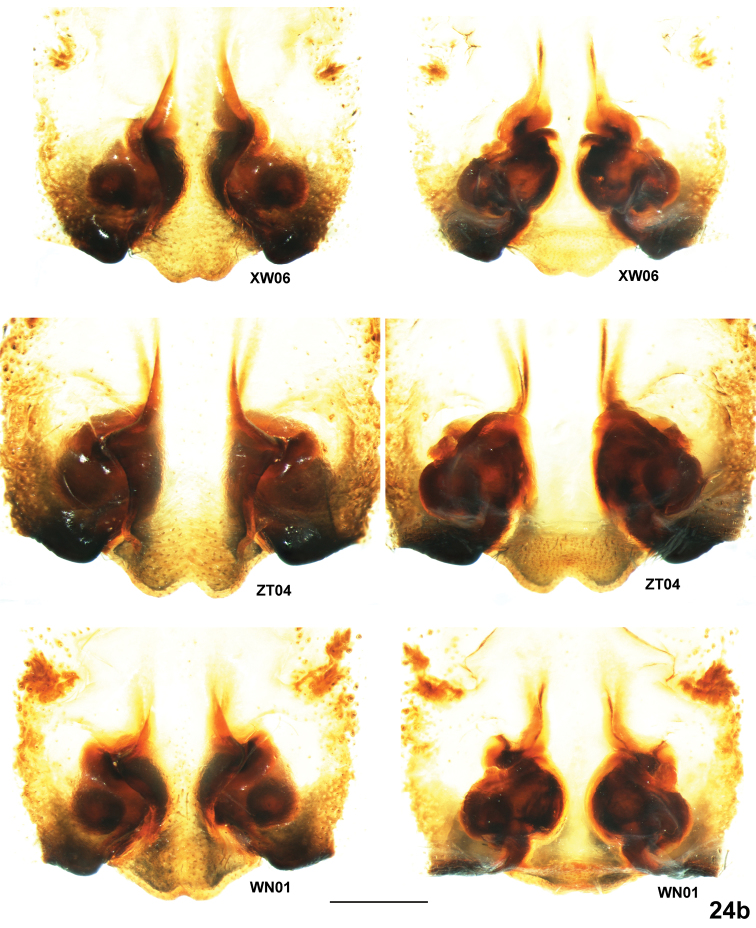
*Psechrus kunmingensis* Yin, Wang & Zhang, 1985. Intraspecific variation of female copulatory organ (left row, epigyne, ventral; right row, vulva, dorsal). (Scale bar, 0.5mm).

Note. Illustrations given in previous publications about *Psechrus kunmingensis* were wrong with the arising position of the spermathecal heads or the position of the spermathecae. Yin et al. (1985, 25, Fig. 5A–D) and Song and Chen (1999, 397 Fig. 232 C–D) were almost the same. They mis-illustrated the spermathecal heads arise posteriorly. The spermathecae are at lateral vulva not medial vulva as shown by Wang and Yin (2001, 334, Figs 9–10).

Spination of palp and legs as shown in Table 1; Measurements of the palp and legs as shown in Table 2. KM37 first, those of others given as ranges in parentheses. Leg formula: 1423.

#### Intraspecific variation.

All 6 examined males were collected from the type locality. The distal fork of EA is relatively variable (Fig. 23): one male (KM18) with 2 short and tiny apophysis, some with relatively strong and blunt forks (KM01, KM04 and KM38), while others exhibit slender and sharp forks with the ventral one curved distally (KM02 and KM03).

All examined adult females vary in many aspects (Figs 10–11, 24a, 24b): 1) the number of SO varies from five (KM05, ZT01, HN01 and WN01) to seven (KM37 and KM36); 2) incision at posterior margin of MS is less distinct in some individuals (XW06, WN01, LX03 and KY02), but others are evident and symmetrical (KM37, HN01 and ZT04); The epigyne of female specimens collected from Zhaotong (the northernmost locality) to Kaiyuan (the southernmost localiy) show disciplinary changes: the lateral bulges of MS range from very sharp (ZT04) to really broadly rounded (KY02); the CD vary from broad and distinctly visible (KM37, HN01, LX03 and KY02 ) to almost invisible (WN01 and ZT04); almost the whole spermathecae are covered (KM37, XW06, ZT04) to more than half part spermathecae are exposed (HN01, LX03 and KY02); 6) spermathecal heads show huge variation in shape and length, and among the dissected vulvae, no two are exactly the same, even opposing sides of the same individual.

#### Distribution.

**Figure 25.**
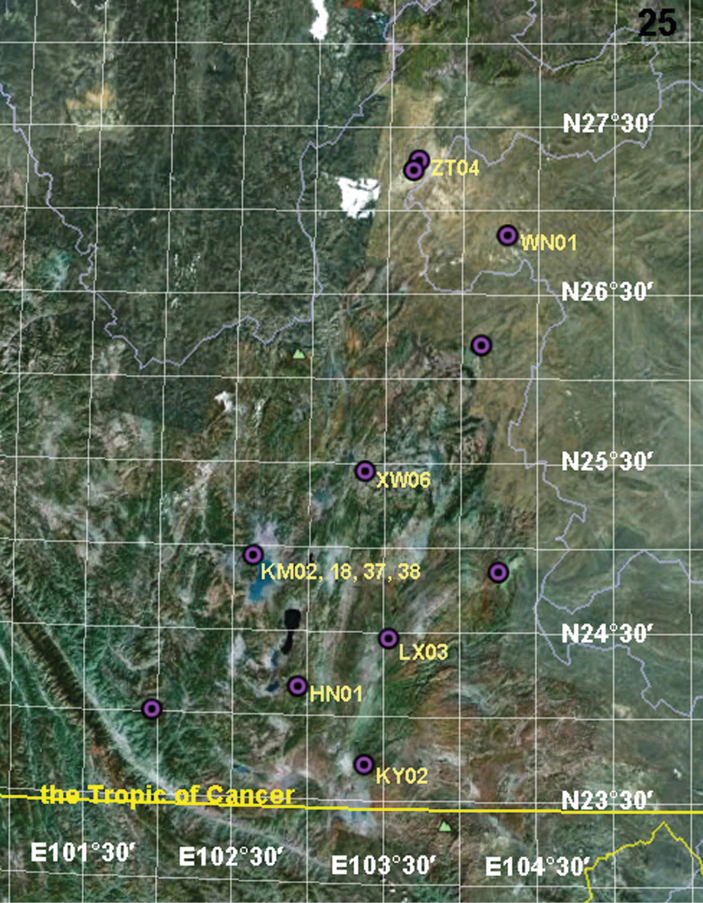
Known collecting localities of *Psechrus kunmingensis* Yin, Wang & Zhang, 1985. The labels near the dots indicate the specimens those shown in the above figure plates.

CHINA: Yunnan Province (Kunming, Qujing, Zhaotong, Yuxi, Honghe), Guizhou Province (Bijie), see Fig. 25.

## Supplementary Material

XML Treatment for Psechrus kunmingensis
